# Effective Control of *Salmonella* Enteritidis in Poultry by Dietary Supplementation with Microencapsulated Essential Oils

**DOI:** 10.3390/antibiotics14060552

**Published:** 2025-05-29

**Authors:** Heitor Leocádio de Souza Rodrigues, Valdinete Pereira Benevides, Isis Mari Miyashiro Kolososki, Mauro M. S. Saraiva, Nayla Pádua Del Bianco Gontijo Souki, Tarley Araújo Barros, André Luis Costa Rabelo, Viviane Amorim Ferreira, Melissa Freitas Feitosa Dix, Adriana Maria Almeida, Cesar Augusto Roque-Borda, Angelo Berchieri Junior

**Affiliations:** 1 School of Agricultural and Veterinary Sciences, São Paulo State University (UNESP), Jaboticabal 14884-900, Brazilvaldinete.benevides@unesp.br (V.P.B.);; 2 Agroceres Multimix Animal Nutrition, Rio Claro 13502-741, Brazil; 3 Vicerrectorado de Investigación, Universidad Católica de Santa Maria, Arequipa 04000, Peru

**Keywords:** cinnamon, clove, oregano, phytobiotics, poultry farm, synergistic effect

## Abstract

**Background/Objectives: ***Salmonella enterica* subsp. *enterica* serovar Enteritidis (*S.* Enteritidis) is a major pathogen associated with poultry products, and the rise of antimicrobial-resistant strains has intensified the need for effective natural control strategies. Essential oils (EOs) are recognized for their antimicrobial potential, but their volatility, instability, and risk of toxicity at high concentrations limit their practical application. Therefore, the objective of this study was to evaluate the antimicrobial efficacy of EOs in broilers infected with *S.* Enteritidis and to characterize potential synergistic or antagonistic interactions between the oils. **Methods**: To achieve this, the oils were first assessed through Minimum Inhibitory Concentration (MIC), Minimum Bactericidal Concentration (MBC), and Fractional Inhibitory Concentration (FIC) assays, and the most effective ones against *S*. Enteritidis were selected. These selected oils were then microencapsulated and incorporated into the broiler feed for the in vivo assay. **Results**: The encapsulated formulation retained key bioactive compounds and significantly reduced bacterial shedding and intestinal colonization when administered to broilers experimentally infected with *S*. Enteritidis. Broilers receiving the optimized half-dose supplementation exhibited a 36% reduction in fecal shedding and a 4 log_10_ decrease in cecal bacterial counts compared to untreated controls. A transient reduction in liver colonization was also observed, while feed intake remained unaffected. **Conclusions**: These findings demonstrate that microencapsulated EOs can serve as an effective natural strategy to control *S.* Enteritidis in poultry. The results support the broader application of lipid-based encapsulation technologies for improving the functional performance of phytobiotics in animal production.

## 1. Introduction

*Salmonella enterica* subsp. *enterica* serovar Enteritidis (*S*. Enteritidis) is among the most frequently isolated paratyphoid serovars in poultry products and human foodborne infections [1]. Although infection in poultry is often asymptomatic, the bacterium is still excreted and disseminated within and beyond the poultry environment [2]. In addition to its rapid spread, antimicrobial-resistant or multidrug-resistant *Salmonella enterica* strains are frequently isolated from poultry production, likely due to the indiscriminate use of antimicrobials, posing a challenge to One Health [3,4,5].

Given the need to control the spread of *Salmonella* strains and the restrictions on conventional antimicrobials in poultry farming, novel antimicrobial products have been investigated worldwide. Among the natural alternatives, essential oils (EOs) have been proposed due to their reported antimicrobial activity against various avian paratyphoid *Salmonella* strains [6,7]. Different applications of these phytobiotics have been suggested in poultry farming, including carcass decontamination [8], performance enhancement in broilers [9], litter sanitization [10], egg disinfection [11], and antimicrobial agents to control *Salmonella* in poultry [12]. Although their precise mechanisms of action have not been fully elucidated, the antimicrobial activity of EOs is hypothesized to be associated with their interaction with the bacterial cell wall [13,14]. This effect may be even more potent when EOs are synergistically combined with conventional antimicrobials or other EOs, enhancing the efficacy of one or both agents, such as tetracycline and thyme [15].

Some limitations associated with the use of EOs as antimicrobials in poultry include high volatility, sensitivity to oxygen and/or luminosity, low water solubility, and rapid degradation in the upper gastrointestinal tract [16]. To mitigate these challenges, EO encapsulation technologies have been proposed as a promising approach to reduce volatilization, enhance bioavailability, and improve the delivery of these compounds within the host organism [17]. Microencapsulation allows the active compounds to be embedded within a protective matrix, reducing evaporation and degradation while facilitating controlled release at target sites, such as the intestinal tract [18].

Lipid-based matrices have shown favorable physicochemical compatibility with EO components, making them suitable carriers for feed applications [19,20,21,22]. Additionally, encapsulation can enhance storage stability, mask strong odors, and minimize adverse effects related to EO overdosing [23,24,25]. In this context, the present study aimed to evaluate the antimicrobial efficacy of microencapsulated EOs against *S.* Enteritidis in experimentally infected poultry and characterize the occurrence of synergism or antagonism among phytobiotics in vitro.

## 2. Results

### 2.1. In Vitro Results

Among the EOs evaluated, cinnamon EO exhibited the strongest antimicrobial activity against *S.* Enteritidis, with minimum inhibitory concentration (MIC) and minimum bactericidal concentration (MBC) values of 0.22 and 0.44 mg/mL, respectively. Oregano (MIC: 0.49 mg/mL), white thyme (0.57 mg/mL), clove (0.69 mg/mL), and mentha (0.69 mg/mL) also demonstrated significant inhibitory effects. In contrast, no inhibitory activity was detected for black pepper, ginger, copaiba, or sweet pear orange EOs, even at a maximum tested concentration of 10% (*v*/*v*) (Table 1).

Bactericidal activity, as indicated by MBC values, varied among the EOs. While cinnamon, oregano, and tea tree oils eliminated the pathogen at relatively low concentrations, other EOs—including basil, mentha, clove, white thyme, and eucalyptus—required concentrations four times higher than their respective MICs to achieve the same effect. These findings underscore a diverse range of bactericidal potential among the tested oils. All MIC and MBC values are summarized in Table 1.

In addition to individual efficacy, selected EOs were evaluated in combination to determine potential interactive effects against *S.* Enteritidis. Among the top-performing EOs—cinnamon, clove, oregano, white thyme, and mentha—synergistic, commutative, indifferent, and antagonistic interactions were observed, as detailed in Table 2. A synergistic interaction was identified between mentha and oregano, where the MIC values of mentha and oregano were reduced by four- and two-fold, respectively, yielding a Fractional Inhibitory Concentration Index (FICI) of 0.75. In contrast, combinations of mentha with white thyme or cinnamon resulted in antagonistic effects, with FICI values of 8 and 6, respectively, and a four-fold increase in their MICs related to the individual oils. Several pairings—including oregano/cinnamon, oregano/white thyme, cinnamon/clove, white thyme/clove, and mentha/clove—exhibited commutative effects (FICI = 2), indicating additive but non-synergistic interactions. The combination of oregano and clove was classified as indifferent (FICI = 1), suggesting that the combined effect was neither enhanced nor diminished relative to the individual activities.

### 2.2. Encapsulation and Quantification of Active Compounds

Following the identification of synergistic effects between mentha and oregano EOs, a lipid-based encapsulation system was developed to enhance the stability of the bioactive compounds. For this, a hydrophobic matrix was employed to encapsulate a blend composed of cassia cinnamon, oregano, and clove EOs. The selected mass proportions were 92.26 g (cassia cinnamon), 104.84 g (oregano), and 146.77 g (clove), totaling 343.9 g of EOs. The encapsulation matrix consisted predominantly of fat (86%), with the EO blend comprising 11% of the total mass and silica acting as an adsorbent at 3%. The final powdered product reached a total mass of 3194.88 g. This approach aimed to protect the active compounds from volatilization and degradation.

The selection of cassia cinnamon, oregano, and clove EOs for encapsulation was based on their individual antimicrobial activity against *S.* Enteritidis, the chemical stability of their major constituents, and their compatibility with lipid-based matrices. Although the combination of mentha and oregano demonstrated in vitro synergism, mentha EO was excluded from the encapsulated formulation due to its antagonistic interaction with cassia cinnamon and white thyme EOs.

The average particle diameter of the microcapsules was 28.34 ± 2.65 µm (*n* = 20), based on the mean of two perpendicular measurements per capsule. The observed values ranged from 25.45 to 33.81 µm. These results indicate a relatively uniform size distribution of the lipid-encapsulated EO particles (Figure S1).

**Figure 1:**
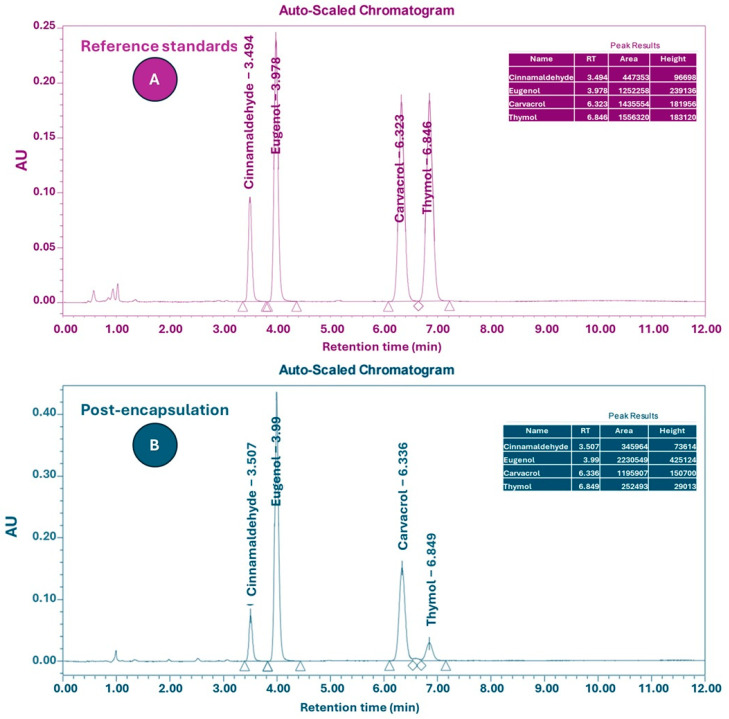
(**A**) HPLC retention profiles of analytical markers: cinnamaldehyde, eugenol, carvacrol, and thymol. (**B**) Chromatographic profile of the encapsulated EO blend analyzed using HPLC. Detection performed at 203 nm; major peaks correspond to retained active compounds within the lipid matrix.

High-Performance Liquid Chromatography (HPLC) analysis was performed to assess the retention of active constituents’ post-encapsulation (Figure 1). The quantified markers included carvacrol and thymol (from oregano oil), eugenol (from clove oil), and cinnamaldehyde (from cinnamon oil). In the unencapsulated blend, the concentrations of carvacrol, thymol, eugenol, and cinnamaldehyde were 20.7%, 3.98%, 35.3%, and 19.9%, respectively (Table 3). Following encapsulation, the corresponding values decreased to 2.02%, 0.39%, 3.49%, and 1.99%, reflecting an approximately 10-fold dilution. It is important to clarify that the observed decrease in the concentrations of carvacrol, thymol, eugenol, and cinnamaldehyde post-encapsulation (approximately 10% of their initial values) reflects the dilution effect resulting from the incorporation of the essential oil blend into a much larger lipid matrix. This reduction does not represent compound degradation or poor recovery during encapsulation. Due to confidentiality agreements with the encapsulating company, quantitative recovery data (e.g., encapsulation efficiency) could not be disclosed. However, the consistent biological activity observed in vivo indicates that a functionally relevant proportion of the active compounds was retained and effectively delivered. This is consistent with previous reports describing encapsulation efficiencies ranging from 20% to 85% for lipid-based systems, depending on formulation strategies and analytical conditions.

Due to the different dilution factors applied to the sample and standards, the peak areas were not directly proportional. Therefore, the final percentages shown in Table 3 reflect concentration values corrected by both the dilution factors and standard curves, rather than raw or normalized peak area ratios.

Based on the concentration of active compounds in the encapsulated product, the recommended inclusion level in animal feed was calculated as 41 g/kg. This corresponds to 1.1 g of cinnamon oil, 1.25 g of oregano oil, and 1.75 g of clove oil per kg of feed, ensuring delivery of 4.1 g of the EO blend per kg. The encapsulated product was protected from light, heat, and moisture to maintain stability until use.

### 2.3. Chick In Vivo Results

To investigate the in vivo antimicrobial efficacy of the microencapsulated EO blend, a broiler infection model was used, involving oral challenge with *S*. Enteritidis. The EO blend—comprising cassia cinnamon (1.1 g/kg), clove (1.75 g/kg), and oregano (1.25 g/kg)—was administered in feed at two concentrations. HPLC analysis previously confirmed that encapsulation retained approximately 10% of the initial concentration of active compounds (see Section 2.2).

**Figure 2:**
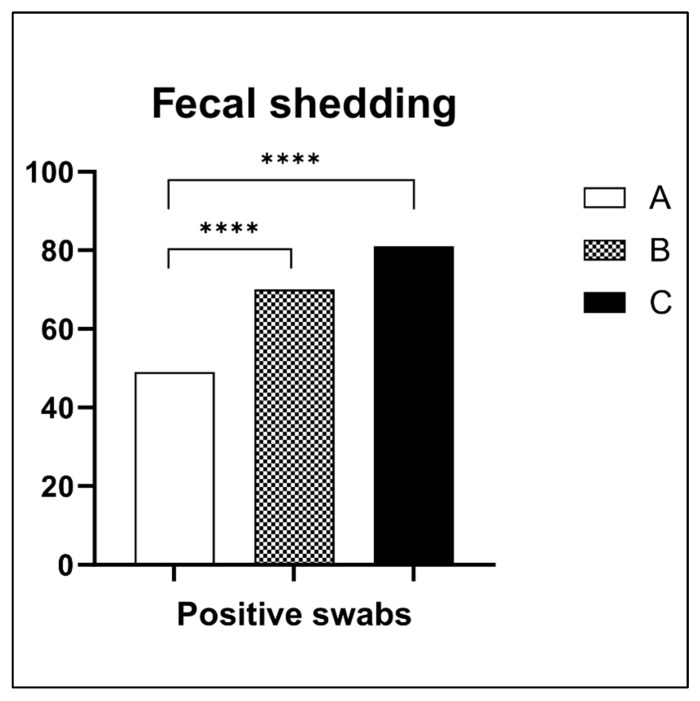
Fecal shedding of *S.* Enteritidis during 21 days of the experiment. Groups A (half dose of EO blend), B (full dose of EO blend), and C (non-treatment). A = 54.44% positive swabs for *S.* Enteritidis; B = 77.77%; C = 90%. **** High statistical difference between groups A/B and A/C.

Fecal Shedding—Cloacal swab analysis revealed significant differences in *S.* Enteritidis fecal excretion across groups (Figure 2). Group A exhibited a 54.44% positivity rate (49/90), substantially lower than the 77.77% in group B (70/90) and 90% in group C (81/90). The 36% fewer positive swabs from fecal excretion in group A relative to the control was statistically significant (*p* < 0.05), indicating that the lower EO dose was more effective at limiting pathogen shedding.

**Figure 3:**
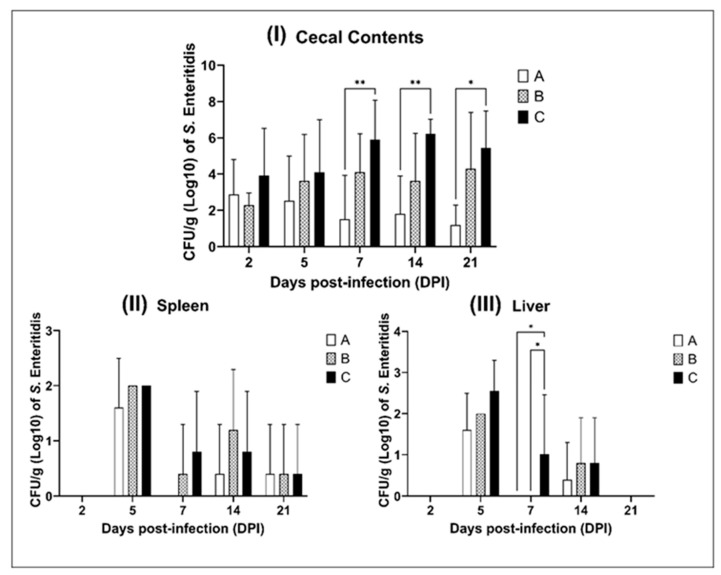
The viable CFU/g of cecal contents from chickens inoculated with *S.* Enteritidis (SE P125 109): (**I**); spleen (**II**); and liver (**III**). Group A (half dose of EO blend), group B (full dose of EO blend), and group C (non-treatment). * (*p* < 0.05); ** (*p* < 0.01); Statistical difference in comparison between groups A, B, and C using two-way ANOVA with Bonferroni’s Multiple Comparisons test at 5% probability.

Cecal Colonization—Bacterial enumeration in cecal contents (Figure 3I) confirmed the superior effect of the lower EO dose. At 7 dpi, group A chicks presented a significant 4 log_10_ reduction in *S.* Enteritidis counts compared to group C (*p* < 0.01). This effect remained statistically significant at 14 dpi (*p* < 0.01) and persisted through 21 dpi (*p* < 0.05), demonstrating a sustained inhibitory effect of the microencapsulated EOs on intestinal colonization. In contrast, group B showed no significant improvement compared to the control.

Systemic Dissemination: Liver and Spleen—Bacterial translocation to systemic organs was evaluated by enumerating CFUs in liver and spleen samples (Figure 3II,III). In the spleen, no significant differences in bacterial load were observed among groups at any time point. In the liver, however, a notable transient effect was detected at 7 dpi. Both groups A and B demonstrated significantly lower *S.* Enteritidis counts compared to group C (*p* < 0.05). While the reduction was more pronounced in group A, no significant differences were observed among groups at 14 and 21 dpi.

**Figure 4:**
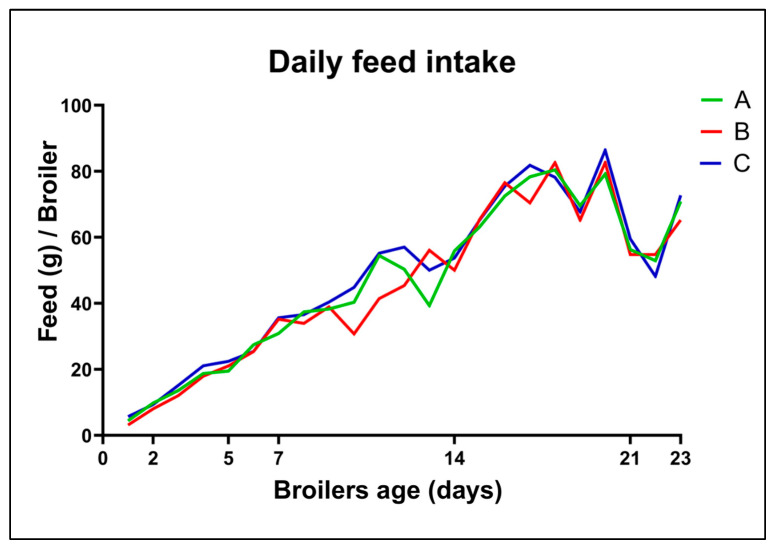
Average daily feed intake of broilers of groups A (half dose of EO blend), B (full dose of EO blend) and C (non-treatment).

Daily feed Intake—Daily feed intake measurements (Figure 4) revealed no statistically significant differences between treated and untreated groups throughout the 23-day experiment. All groups followed a similar feed intake trajectory, indicating that the inclusion of the EO blend, regardless of concentration, did not impair feed palatability or broiler appetite. This supports the potential feasibility of EO inclusion in poultry diets from a production standpoint.

Although no histological evaluation was performed, necropsy findings provided valuable insights into tissue-level responses. Macroscopic examination of all broilers from group B (7–14 dpi) revealed signs of intestinal mucosal congestion, hepatic discoloration, and yolk sac retention in several animals. These lesions were not observed in group A and were minimal in group C.

## 3. Discussion

EOs derived from culinary herbs—such as cinnamon, clove and oregano — have long been utilized for their antimicrobial properties, particularly in food preservation and safety applications [26]. Their increasing use as phytobiotics in poultry production aims to control enteric pathogens, such as *Salmonella enterica*, while minimizing antibiotic reliance [16]. However, their practical use is often limited by volatility, poor solubility, and challenges in maintaining effective concentrations at target sites. Microencapsulation technologies have emerged as a strategic solution, allowing gradual release along the gastrointestinal tract and improved systemic bioavailability [27]. This is especially relevant considering that non-encapsulated EO treatments often show lower reductions in cecal and cloacal colonization by *Salmonella* [12,28].

Our results confirm the in vitro activity of certain EOs against *S.* Enteritidis, with cinnamon EO demonstrating the strongest inhibitory and bactericidal activity (MIC = 0.22 mg/mL). This result aligns with previous findings attributing cinnamon’s efficacy to cinnamaldehyde, a major active compound known to compromise bacterial membrane integrity and modulate gene expression [7,16,29]. Oregano, white thyme, mentha, and clove also displayed relevant antimicrobial effects, in line with reports documenting their phenolic components—such as thymol, carvacrol, menthol, and eugenol—as responsible for disrupting bacterial homeostasis [30]. In contrast, black pepper, ginger, copaiba, and sweet pear orange EOs exhibited no inhibitory activity against *S.* Enteritidis under the tested conditions, despite previous reports of their antimicrobial efficacy against other pathogens [8,31]. These discrepancies highlight the strain-specific nature of EO activity, which can be influenced by factors such as bacterial membrane composition, pH, emulsification capacity, and oil solubility [32].

The bactericidal activity profile revealed that all inhibitory EOs could eliminate *S.* Enteritidis at concentrations ranging from 1× to 4× MIC, consistent with earlier studies showing similar MIC–MBC relationships for *Salmonella* spp. [6,33,34]. This suggests that although some EOs act as bacteriostatic agents at low concentrations, increasing the dose can shift their activity toward bactericidal effects, an important consideration for dosage optimization in feed applications. Moreover, we highlight that the study’s strength lies in the combinatorial evaluation of EOs interactions through FICI analysis — an approach seldom addressed in poultry or veterinary microbiology trials despite the common use of EO blends — and, notably, a synergistic interaction was observed between mentha and oregano EOs, resulting in substantial MIC reduction.

On the other hand, antagonistic effects were observed in combinations such as mentha with white thyme or cinnamon, where the MICs of both components increased significantly. This highlights a critical but often overlooked issue: the empirical mixing of EOs without mechanistic compatibility testing may lead to reduced effectiveness, or even counterproductive interactions. This reinforces prior warnings against arbitrary EO combinations [16] and emphasizes the need for rational formulation design; also, the indifferent interaction observed between oregano and clove (FICI = 1) and the predominance of commutative (additive) effects further underscore the complexity of EO interactions. Interestingly, while clove and cinnamon exhibited additive behavior in this study, previous research has shown them to be synergistic or antagonistic depending on the target microorganism [35,36]. For instance, synergy was reported against *S.* Typhimurium and *Aspergillus niger*, whereas antagonism was noted against *Escherichia coli* but not *Listeria monocytogenes* or *Yersinia enterocolitica*. These observations support the notion that EO interactions are highly context-dependent, influenced by microbial physiology, EO composition, and testing conditions [37], which can pose difficulty in the use of EOs. Overall, the initial findings of this study provide mechanistic and practical insights into the antimicrobial potential of individual and combined EOs. While some combinations can significantly enhance activity through synergism, others may compromise efficacy due to antagonistic interactions.

Although mentha EO demonstrated in vitro synergism with oregano, it was excluded from the final EO blend due to antagonistic interaction with cinnamon. This decision was based on preliminary FICI results that indicated a reduced antimicrobial effect when mentha and cinnamon were combined. Nevertheless, this exclusion may have overlooked potentially effective combinations, such as mentha + oregano without cinnamon. Future studies should consider testing alternative binary or ternary EO blends to further optimize antimicrobial efficacy while avoiding antagonistic interactions.

The encapsulation approach applied in this study represents a critical technological intervention to enhance the usability of bioactive compounds in animal nutrition and food science [38]. By embedding the bioactive compound blend into a lipid or polymeric matrix, the strategy aimed not only to reduce volatility and degradation but also to enable site-specific release in the intestinal tract—a key requirement for targeting enteric pathogens such as *Salmonella* sp. [18,39,40]. Rather than focusing on the quantitative loss during encapsulation—an inherent characteristic of the dilution process—this study demonstrates that biological efficacy can still be preserved even when active compound concentrations are significantly reduced. Although we did not assess the controlled release of the EO blend in this study, previous research using similar capsules has reported that their degradation occurs gradually along the gastrointestinal tract [23,24,25]. The observed antimicrobial performance in vivo, particularly in group A, supports the hypothesis that controlled release and local delivery may compensate for lower nominal concentrations by improving compound stability and bioaccessibility at the site of infection [41].

The formulation, based on hydrogenated vegetable fat and silica, enables cold dispersion in feed mixtures and offers protection during storage and thermal processing, and these attributes are particularly relevant for industrial-scale feed production, where compound volatility and oxidative instability typically limit the phytogenic use [42]. Although the process preserved four key analytical markers (carvacrol, thymol, cinnamaldehyde, and eugenol), it is plausible that minor volatile or synergistic compounds were lost or chemically altered during encapsulation, potentially modifying the overall spectrum of activity [43]. In the present study, the microcapsules produced had an average diameter of 28 µm, which is considerably smaller than those reported in previous research [38,44], where capsule sizes reached approximately 32–2000 µm for poultry administration. Smaller particle sizes, such as those obtained in our formulation, may offer advantages in terms of more uniform blending into feed and easier ingestion by broilers, potentially improving the distribution and bioavailability of the active compounds [45,46]. Although exact encapsulation efficiency values could not be disclosed due to confidentiality agreements with the partnering company, the observed antimicrobial efficacy in vivo supports the hypothesis that the major bioactive compounds were adequately preserved and delivered. The apparent reduction in compound concentration to approximately 10% of the original values was due to intentional dilution in the lipid matrix, not compound loss. This aligns with previous reports showing encapsulation recoveries ranging from 20% to 85% in lipid- and polymer-based systems [23,24,25,38,44], indicating that the formulation applied here falls within the expected range for effective encapsulation in animal feed applications.

For the in vivo trial, a full dose of EOs was selected compared to those typically reported in poultry studies [9,12,47,48] to evaluate anti-*Salmonella* efficacy at elevated doses. While most studies focusing on growth performance use EO blends at 150–800 mg/kg [9,12], antimicrobial effects have been observed with much lower concentrations, such as 50–100 mg/kg of cinnamaldehyde or thymol, which reduced *S.* Enteritidis prevalence by up to 25% [49]. Another study combining 45 mg/kg of carvacrol, 100 mg/kg of cinnamaldehyde, 200 mg/kg of cineole, and 2 g/kg of capsaicin achieved a 3 log reduction in *S.* Enteritidis [28]. These findings suggest that concentrations ranging from 100 to 600 mg/kg per EO may be sufficient for pathogen control in poultry, although outcomes may vary depending on environmental challenges and the bioavailability of the active compounds in the host organism [50].

In our study, supplementation with the EO blend significantly reduced fecal shedding of *S.* Enteritidis by approximately 36% compared to the untreated control. This result is consistent with previous reports showing reductions in the shedding of *S.* Heidelberg, *S.* Minnesota, and *S.* Typhimurium following treatment with EOs and organic acids [51]. These findings underscore the potential of EO-based strategies to decrease horizontal transmission of paratyphoid *Salmonella* serovars in poultry production systems. A notable finding was the significant 4 log_10_ reduction in cecal *S.* Enteritidis counts observed in broilers treated with half the recommended EO dose. This reduction was evident from 7 dpi and persisted through 21 dpi. Similar time-dependent antimicrobial effects have been reported in other studies, including reductions observed at 7 and 14 dpi [51] and even earlier, as reported by Hu et al. [12], where declines began at 3 dpi and extended to 10 dpi. These results reinforce the importance of defining an optimal dose–response curve to guide the future standardization of EO-based antimicrobials for poultry applications. Although the EO blend significantly reduced cecal bacterial load, no significant changes were observed in spleen colonization. However, a transient decrease in liver colonization was noted at 7 dpi in the treated groups. This may reflect a reduction in the translocation of bacteria from the intestine to systemic organs, possibly due to improvements in gut integrity and microbial balance promoted by EOs [12].

Interestingly, the most pronounced antimicrobial effect was observed at the lower dose of the EO blend. This paradox may be explained by the non-selective antimicrobial properties of EOs, which, at higher concentrations, could disrupt the intestinal microbiota and damage the mucosal barrier. Supporting this hypothesis, Laptev et al. [52] reported marked shifts in the gut microbiota of EO-treated chickens infected with *S.* Enteritidis, including reductions in beneficial genera such as *Bifidobacterium* and *Lactobacillus*. These findings suggest that high EO doses may inadvertently compromise gut health and indicate potential dose-dependent toxicity associated with higher EO concentrations, aligning with previous reports of mucosal irritation or microbiota disruption linked to phenolic EO components [53,54,55]. The lower effectiveness and apparent adverse effects in group B may reflect the non-selective antimicrobial nature of the blend at excessive doses, potentially leading to gut dysbiosis or epithelial damage [56]. Daily feed intake remained unaffected by EO supplementation, indicating good palatability and compatibility of the blend at both tested concentrations. Similar results have been reported in the literature [9,57]. Although this study did not include zootechnical performance assessments due to the infection-focused design, prior research has shown that EO supplementation can improve the feed conversion ratio, enhance gut morphology, and reduce carcass fat content in broilers [9,58].

A key limitation of this study is the extrapolation of in vitro findings to in vivo conditions, which involve complex physiological variables such as digestive enzymatic activity, pH fluctuations, and interactions with the gut microbiota—factors that can significantly influence the efficacy of EOs. Moreover, the observed signs of mucosal and hepatic damage in broilers administered the higher EO dose highlight the importance of comprehensive toxicity assessments. Additional studies are needed to clarify the mechanisms of action of the encapsulated compounds; verify their systemic bioavailability, degree of homogenization of EO blends in poultry feed, and encapsulation efficiency; and evaluate their safety across a range of physiological conditions. Future research should also address the stability of the microcapsules during feed processing and storage, as well as their scalability and cost-effectiveness for commercial applications. Despite these limitations, the present findings remain robust and support the conclusion that lipid-encapsulated EO blends—when administered at appropriate doses—represent a promising, natural, and effective alternative to conventional antimicrobials for *Salmonella* control in poultry production systems.

## 4. Materials and Methods

### 4.1. Chemical Reagents

Mueller–Hinton Broth (MHB) was purchased from Oxoid (Hampshire, UK), and Brilliant Green Agar (BGA) from Kasvi (Spain). Selenite broth was obtained from HiMedia (Mumbai, India), and Luria–Bertani (LB) broth from Sigma-Aldrich (St. Louis, MO, USA). Analytical standards, including carvacrol (W224502), thymol (T0501), cinnamaldehyde (C80687), and eugenol (E51791), were also obtained from Sigma-Aldrich (St. Louis, MO, USA). Phosphate-buffered saline (PBS, pH 7.2) was prepared using analytical-grade reagents.

### 4.2. Essential Oils

Sixteen EOs were purchased from Destilaria Bauru^®^ (São Paulo, Brazil), and detailed specifications from each oil, such as origin and plant species, are arranged in Table 4 (extracted by steam distillation). For characterization, 1 mL aliquots of each EO were placed into individual Eppendorf tubes and weighed to determine their densities (mg/mL). EO aliquots (1 mL each) were individually placed into Eppendorf tubes, weighed to determine their densities (mg/mL), and stored under refrigeration (<5 °C) until the preparation of stock solutions. Stock solutions were initially prepared at a 1% EO (*v*/*v*) concentration, containing 1% Tween 80 as an emulsifier, with PBS (pH 7.2) completing the final volume; for EOs lacking antimicrobial activity at 1%, the concentrations were increased up to 10% EO. Finally, the stock solution was homogenized using a vortex mixer at 3800 rpm for five minutes to promote oil emulsification.

### 4.3. In Vitro Studies

#### 4.3.1. Bacterial Strain

For this study, we selected an *S.* Enteritidis strain (SE P125 109) originally isolated from contaminated eggs and associated with human salmonellosis outbreaks in England. An overnight culture of *S*. Enteritidis was prepared by incubating 10 mL of MHB at 37 °C with orbital agitation at 150 rpm. After incubation, the bacterial suspension was diluted in phosphate-buffered saline (PBS, pH 7.2) and adjusted to approximately 10^33^ colony-forming units (CFU/mL) for use in vitro assays. All experimental procedures were carried out at the Department of Pathology, Reproduction and One Health (DPRSU), School of Agricultural and Veterinary Sciences, São Paulo State University (FCAV/Unesp).

#### 4.3.2. Antimicrobial Activity

MIC was performed in sterile 96-well microplates following the Clinical and Laboratory Standards Institute guidelines [59], with minor adaptations based on Munive Nuñez et al. [60]. Briefly, 95 µL of MHB was added to each well, then 100 µL of the EO stock solution was added to the first well of each row, except for the positive control, which contained MHB and bacterial suspension only. Using a multichannel micropipette, we slowly homogenized the stock solution into the MHB. After homogenization, a two-fold serial dilution was performed by transferring 100 µL from the first to the subsequent wells. Next, 5 µL of the standardized bacterial suspension was added to all wells, except for the negative control (MHB + EO only), resulting in a final volume of 100 µL per well. Plates were incubated at 37 °C for 24 h. After incubation, 30 µL of 0.01% resazurin solution was added to each well, and results were obtained after 30 min, based on the visual reading of the colorimetric reaction occurrence, where blue indicated bacterial inhibition and pink indicated the absence of inhibition. The MIC was defined as the lowest EO concentration at which the well maintained a blue color.

For MBC, 10 µL measures from the last four wells showing no visible growth in the MIC assay were spot inoculated onto BGA plates, as described by Munive Nuñez et al. [60]. Plates were incubated at 37 °C for 24 h, and the MBC was defined as the lowest concentration at which no bacterial colonies were observed.

#### 4.3.3. Synergism Assessment of Essential Oils

To evaluate the synergistic interactions between EOs, a Checkerboard assay was conducted using a 6 × 6 microplate format. Five EOs exhibiting the lowest MIC values against *S.* Enteritidis were selected, resulting in ten possible combinations. Stock solutions were individually prepared for each oil at sixteen times their respective MIC values (16 × MIC), mixed with 1% Tween 80, and adjusted to final volume with PBS.

**Figure 5:**
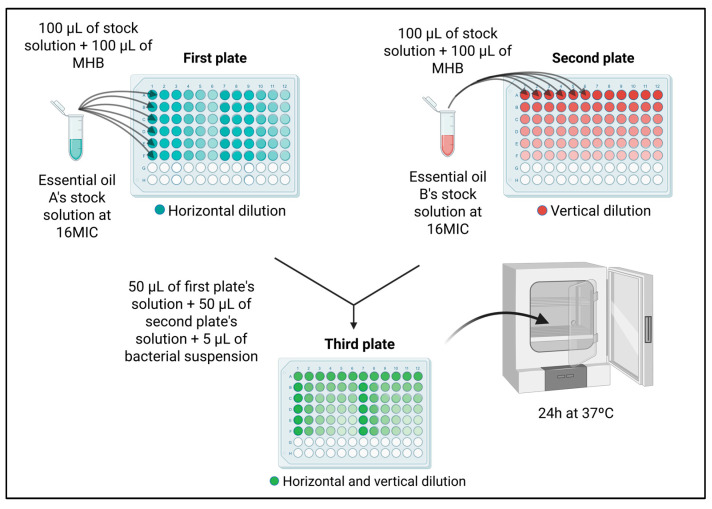
Method of the Checkboard assay. MHB = Mueller–Hinton Broth; MIC = minimum inhibitory concentration. This figure was created using Biorender.com.

For each EO combination, three 96-well microplates were prepared by initially dispensing 100 µL of MHB per well. On the first plate, 100 µL of EO from those exhibiting the lowest MIC values (in stock solution) was added into the first six wells of the initial column. After thorough homogenization, serial dilutions were performed horizontally by transferring 100 µL to subsequent columns. Similarly, on the second plate, 100 µL of another one from the lowest-MIC-value EO stock solution was added to the first six wells of the initial row, followed by vertical serial dilutions transferring 100 µL downward into subsequent rows. Afterwards, 50 µL from each of the first six columns of the first plate and 50 µL from each of the first six rows of the second plate were combined into corresponding wells on a third plate. This ensured that the highest concentrations of both oils were in the first well (top-left), progressively decreasing toward the lowest concentrations in the last well (bottom-right), as depicted in Figure 5. After combining these aliquots, 5 µL of bacterial suspension was added into each well, excluding the negative controls, maintaining identical conditions as previously described for MIC determination.

To validate the EO stock solutions, MIC tests were repeated on separate plates using the same five oil solutions. Checkerboard and MIC assay plates were incubated at 37 °C for 24 h. Following incubation, 30 µL of 0.01% resazurin solution was added to each well to interpret visually bacterial growth inhibition, employing the same criteria as the standard MIC assay. The FICI Index was calculated using Equation (1), where “first EO” and “second EO” denote the MIC values for each EO in the mixture and individually. The calculated FICI values were interpreted as synergistic (*FICI* < 1), indifferent (*FICI* = 1), commutative (1 < *FICI* ≤ 2), or antagonistic (*FICI* > 2), according to Maggio et al. [15].

(1) $$FICI=\frac{{MIC}^{A}mixture}{{MIC}^{A}alone}+\frac{{MIC}^{B}mixture}{{MIC}^{B}alone}$$

### 4.4. Encapsulation Strategy and Analytical Assessment

Of five EOs exhibiting the lowest MIC values, three of them were selected based on their favorable interaction profiles, with no antagonism observed in previous in vitro assessments. These oils were encapsulated within a hydrophobic matrix designed to prevent volatilization and promote the bioavailability of EOs. The formulation strategy considered physicochemical compatibility and additive behavior among components, guided by principles derived from the FICI.

EOs were encapsulated in collaboration with Agroceres Multimix Nutrição Animal Ltda., using hydrogenated vegetable fat as the encapsulation matrix. The process consisted of melting the fat, followed by the incorporation of EOs under continuous stirring until complete homogenization. The mixture was then cooled under controlled agitation, leading to the solidification of the lipid matrix. Agitation continued until a powdered product was obtained. Due to industrial confidentiality agreements, specific formulation details and processing conditions cannot be disclosed.

The particle size of the microcapsules was evaluated using optical microscopy (Olympus, DP73—Tokyo, Japan) with CellSens Standard^©^ software, version 4.2 CS-ST-V4.2 (Olympus LS). Approximately 0.5 g of the encapsulated EO blend was deposited onto clean glass slides and imaged under a 20× objective lens. Two orthogonal diameters (length and width) were measured per capsule to account for possible asymmetry. Measurements were taken directly using the software and expressed in micrometers (µm). A total of 20 individual microcapsules were randomly selected and analyzed.

### 4.5. Analytical Performance

To evaluate the retention and stability of key active compounds, the encapsulated and non-encapsulated oils were analyzed in triplicate using chromatography (HPLC; Waters 2695 Separations Module), coupled with a photodiode array UV–Vis detector (Waters 2998, Photodiode Array Detector). Chromatographic separation was achieved using a C18 column (Agilent Technologies, 150 × 4.6 mm, 5 µm SB-C18) under isocratic conditions, with detection at 203 nm. The column oven was maintained at 45 °C, with a flow rate of 1.0 mL/min and an injection volume of 10 µL. The concentrations of active compounds in the EO blend were determined by integration of the chromatographic peak areas obtained via HPLC, similar to the method described by Wang et al. [61]. Quantification was carried out using external standards for carvacrol, thymol, eugenol, and cinnamaldehyde, with calibration curves prepared from standard solutions at known concentrations. For sample preparation, 103.5 mg of the EO blend was extracted in 25 mL of methanol in an ultrasonic bath for 30 min. A second dilution was performed by adding 0.10 mL of this extract to 10 mL of mobile phase (composed of 50% acetonitrile and 50% Milli-Q water), resulting in a final concentration of 41.4 µg/mL. The standards were prepared independently, with final concentrations of 9.9 µg/mL for carvacrol and thymol, 3.2 µg/mL for eugenol, and 10.5 µg/mL for cinnamaldehyde. Quantification was performed both before and after encapsulation.

### 4.6. Ethical Approval

The in vivo study was reviewed and approved by the Ethics Committee on Animal Experimentation (CEUA) of the School of Agricultural and Veterinary Sciences, São Paulo State University (FCAV/Unesp), under protocol number 2931/2024, on June 16, 2024.

#### 4.6.1. Bacterial Inoculum

To prepare the inoculum, we used the same strain of *S.* Enteritidis (SE P125 109) as in the in vitro assay. This strain is resistant to nalidixic acid and spectinomycin (SE^NalSpcR^). The strain was inoculated in LB broth and incubated at 37 °C for 24 h under constant agitation at 150 rpm. After incubation, the inoculum was diluted 10^3^.

#### 4.6.2. Experimental Design

For this study, 135 one-day-old broiler chicks were obtained from a commercial hatchery. These were randomly distributed into three groups (A, B, and C) of 45 animals each. Poultry in group A received a half dose of the EO blend (205 g/10 kg of feed), poultry in group B received the full dose of the EO blend (410 g/10 kg of feed), and poultry in group C received no treatment (the control group).

Upon arrival, sterile swabs were used to collect samples from the bottom of the transport boxes to confirm that the newly hatched chicks were free of *Salmonella* spp. The poultry chicks were housed in acclimatized rooms, separately in metal cages and provided with water and feed *ad libitum*, following species and lineage-specific recommendations. Poultry treated with the EO blend received the product mixed with feed throughout the experiment. To this end, the EO blend was incorporated into the feed and manually homogenized for approximately ten minutes.

On the second day of age, chicks were inoculated into the crop using oral gavage needles with 0.2 mL *S.* Enteritidis^NalSpcR^ culture containing approximately 1  ×  10^6^ CFU. Twice weekly after the challenge, cloacal swabs were collected from 15 broilers per group to inspect the excretion of *S.* Enteritidis^NalSpcR^. Then, the swabs were placed in tubes containing 3 mL of selenite broth plus 0.4% novobiocin. Samples were then streaked onto BGA plates supplemented with nalidixic acid and spectinomycin (BGA^Nal/Spc^). The plates and swab-containing tubes were incubated at 37 °C for 24 h.

To assess cecal colonization and systemic infection, five broilers per group were euthanized at 2-, 5-, 7-, 14-, and 21-day post-infection (dpi) for *S.* Enteritidis^NalSpcR^ enumeration in the liver, spleen, and ceca. Bacterial enumeration was carried out according to the methodology adopted by Berchieri Junior et al. [62]. The obtained CFU/mL values were converted to Log_10_ for statistical analysis and result interpretation.

To measure daily feed intake, the residual feed volume in the feeders was weighed daily and subtracted from the amount added the previous day at standardized times, ensuring a 24 h evaluation period. Data from all three groups were recorded daily in spreadsheets from the beginning to the end of the experiment.

#### 4.6.3. Statistical Analysis

Statistical analysis was conducted using GraphPad Prism© software, version 10.2.3 for Windows©. Fecal shedding data from the three groups were plotted as positive and negative swabs and compared using the Chi-square test. Log-transformed bacterial counts in the liver, spleen, and ceca were analyzed using a two-way ANOVA, followed by Bonferroni’s multiple comparison test. Data on daily feed intake were analyzed using one-way ANOVA and compared using the Chi-square test. Statistical significance was admitted when the *p*-value was lower than 5% (*p* < 0.05).

## 5. Conclusions

This study provides important insights into the in vitro and in vivo antimicrobial activity of various EOs against *S.* Enteritidis. Our results indicate that microencapsulated cinnamon, clove, and oregano EOs are an effective natural alternative for reducing the fecal excretion of *S.* Enteritidis and its systemic infection in broilers, without affecting the daily feed intake of treated broilers. These findings strongly contribute to the maintenance of the One Health approach and minimizing the emergence of resistant or multidrug-resistant bacteria originating from animal production. We emphasize the need for further research to determine optimal and potentially toxic concentrations for poultry, including the assessment of the effects of these phytobiotics on the gut microbiota and tissue integrity in broiler chickens. Therefore, further research exploring lower concentrations and their effects on infection and poultry’s health is needed to establish optimal therapeutic dosing standards in broiler chickens.

## Figures and Tables

**Table 1:** MIC and MBC values of tested EOs against *S.* Enteritidis.

EOs	MIC (mg/mL)	MBC (mg/mL)
Oregano	0.49	0.99
Cinnamon	0.22	0.44
Clove	0.69	2.76
Bay laurel	4.56	4.56
Citronella	4.48	4.48
White thyme	0.57	2.28
Lemongrass	2.25	2.25
Rosemary	11.52	23.05
Tea tree	1.37	1.37
Black pepper *	-	-
Eucalyptus	5.83	23.32
Ginger *	-	-
Basil	5.79	23.17
Copaiba *	-	-
Sweet pear orange *	-	-
Mentha	0.69	2.77

*: no effect against *S.* Enteritidis was observed at a 10% (*v*/*v*) concentration of EOs.

**Table 2:** Fractional Inhibitory Concentration Index (FICI) and interaction classification of selected essential oil combinations against *S.* Enteritidis.

EOs Combinations	FICI	Classification
Oregano/Cinnamon	2	Commutative
Oregano/White thyme	2	Commutative
Cinnamon/White thyme	4	Antagonistic
Cinnamon/Clove	2	Commutative
Oregano/Clove	1	Indifferent
White thyme/Clove	2	Commutative
Mentha/White thyme	8	Antagonistic
Mentha/Clove	2	Commutative
Mentha/Cinnamon	6	Antagonistic
Mentha/Oregano	0.75	**Synergistic**

**Table 3:** Percentage of active compounds in the EO blend (cinnamon + clove + oregano EO) before and after encapsulation.

Compounds	Before Encapsulation (%)	Post-Encapsulation (%)
Carvacrol	20.7	2.02
Thymol	3.98	0.39
Eugenol	35.3	3.49
Cinnamaldehyde	19.9	1.99

**Table 4:** Characteristics of each essential oil tested in vitro for antimicrobial activity against *Salmonella* Enteritidis.

Common Name	Scientific Name	Botanical Source	Geographical Origin
**Oregano**	*Origanum vulgare*	Leaf	Spain
**Cinnamon**	*Cinnamomum zeylanicum*	Bark	Brazil
**Clove**	*Eugenia caryophyllus*	Flower	Brazil
**Mentha**	*Mentha arvensis*	Leaf	Brazil
**White thyme**	*Thymus vulgaris*	Leaf	India
**Citronella**	*Cymbopogon nardus*	Leaf	Brazil
**Lemongrass**	*Cymbopogon flexuosos*	Leaf	Brazil
**Rosemary**	*Rosmarinus officinalis*	Leaf	Spain
**Tea tree**	*Melaleuca alternifolia*	Leaf	India
**Black pepper**	*Piper nigrum* L.	Fruit	India
**Eucalyptus**	*Eucalyptus globulus*	Leaf	Brazil
**Ginger**	*Zingiber officinale*	Root	China
**Basil**	*Ocimun basilicum* L.	Leaf	India
**Copaiba**	*Copaifera officinalis*	Leaf	Brazil
**Sweet pear orange**	*Citrus sinensis*	Fruit bark	Brazil
**Bay laurel**	*Laurus nobilis*	Leaf	Brazil

## Data Availability

Data are contained within the article and Supplementary Materials.

## Supplementary Materials

The following supporting information can be downloaded at https://www.mdpi.com/article/10.3390/antibiotics14060552/s1: Figure S1: Optical microscopy of the microcapsules present in the EO blend under a 20× objective lens. (A) Microcapsules; (B) Measurement of orthogonal diameters (length and width) using CellSens Standard© software, version 4.2 (CS-ST-V4.2).

## Abbreviations

The following abbreviations have been used in this article:

SE	*Salmonella enterica* subsp. *enterica* serotype Enteritidis
EOs	Essential oils
MIC	Minimum inhibitory concentration
MBC	Minimum bactericidal concentration
FICI	Fractional Inhibitory Concentration Index
CEUA	Ethics Committee on Animal Experimentation
MHB	Mueller–Hinton Broth
LBB	Luria–Bertani Broth
BGA	Brilliant Green Agar
Nal/Spc	Nalidixic acid/Spectinomycin
CFU	Colony-Forming Unit
*v*/*v*	Volume by volume
HPLC	High-Performance Liquid Chromatography
