# Supplementary material for: Effective Control of Salmonella Enteritidis in Poultry by Dietary Supplementation with Microencapsulated Essential Oils
Source: Antibiotics (Basel). 2025 May 29;14(6):552. doi: 10.3390/antibiotics14060552 (PMC12189832; doi:10.3390/antibiotics14060552)
Supplement: Supplementary file 1 [file antibiotics-14-00552-s001.zip › antibiotics-3659024-supplementary.pdf]

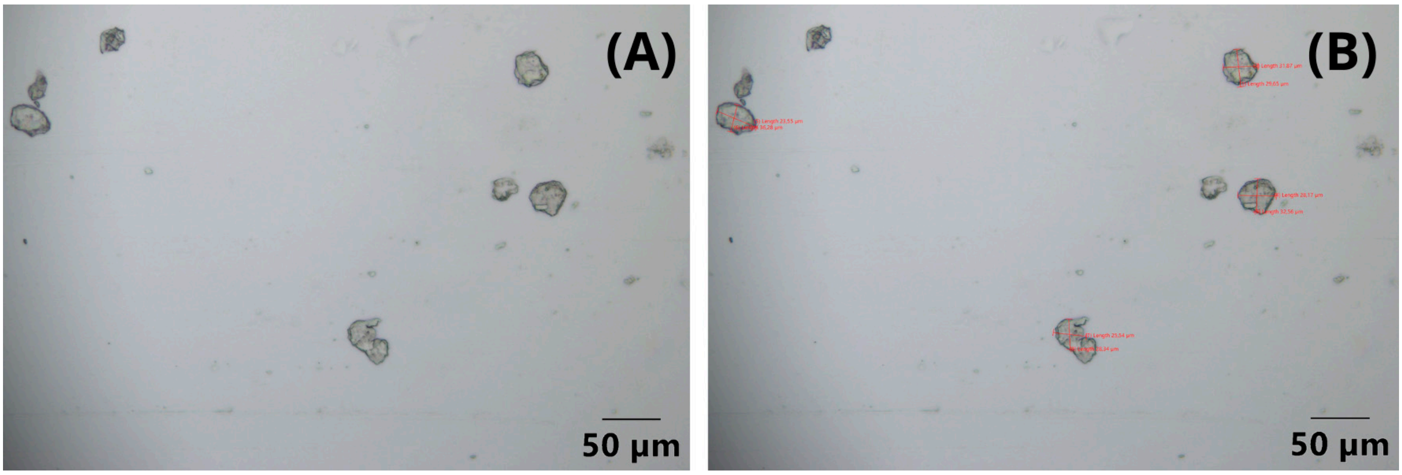


**Figure S1.** Optical microscopy of the microcapsules present in the EO blend under a 20× objective lens. (A) Microcapsules; (B) Measurement of orthogonal diameters (length and width) using CellSens Standard© software, version 4.2 (CS-ST-V4.2).
